# Prevalence, Knowledge and Attitudes Concerning Dietary Supplements among a Student Population in Croatia

**DOI:** 10.3390/ijerph15061058

**Published:** 2018-05-23

**Authors:** Sandra Pavičić Žeželj, Ana Tomljanović, Gordana Kenđel Jovanović, Greta Krešić, Olga Cvijanović Peloza, Nataša Dragaš-Zubalj, Iva Pavlinić Prokurica

**Affiliations:** 1 Health Ecology Department, Faculty of Medicine, University of Rijeka, Braće Branchetta 20, 51000 Rijeka, Croatia; anci.tomljanovic@gmail.com; 2 Department of Health Ecology, Teaching Institute of Public Health of Primorsko-goranska County, Krešimirova 52a, 51000 Rijeka, Croatia; gordana.kendel-jovanovic@zzjzpgz.hr (G.K.J.); natasa.dragas-zubalj@zzjzpgz.hr (N.D.-Z.); 3 Department of Food and Nutrition, Faculty of Tourism and Hospitality Management, University of Rijeka, Primorska 42, P.O. Box 97, 51410 Opatija, Croatia; greta.kresic@fthm.hr; 4 Department of Anatomy, Faculty of Medicine, University of Rijeka, Braće Branchetta 20, 51000 Rijeka, Croatia; olga.cvijanovic@medri.uniri.hr; 5 Croatian Centre for Agriculture, Food and Rural Affairs, Gorice 68 g, 10000 Zagreb, Croatia; iva.pavlinic.prokurica@hcphs.hr

**Keywords:** attitude, dietary supplements, knowledge, students

## Abstract

The aim of this study was to determine the prevalence of usage and the knowledge and attitudes towards dietary supplements among medical sciences and nonmedical sciences students from Croatia. The study was conducted based on a questionnaire about dietary supplement usage, knowledge and attitudes. The prevalence of dietary supplement use, among 910 university students was 30.5%. The most-used dietary supplements were vitamins (18.0% in medical sciences students and 9.8% in non-medical sciences students). For all students, the internet (66.1%) was the most common source of information, followed by healthcare professionals (33.2%). The most common reason for taking dietary supplements was to maintain good health (26.4%). Use of the internet rather than health professionals as a trusted information source should be revised among this young population. Supplement intake was significantly associated with body mass index (BMI) (*p* = 0.016) and physical activity (*p* = 0.050). Students with normal BMI (61.5%) and the most physically active students (37.7%) took significantly more dietary supplements. Results of this study could help medicine faculties to improve their curriculum and support the development of public health messages aimed at wise and safe use of dietary supplements.

## 1. Introduction

Adequate diet plays a key role in the maintenance of good health and the prevention of disease, and a well-balanced diet ensures a sufficient amount of nutrients, vitamins and minerals [1]. Inadequate and irregular nutrition, physical inactivity, stress and cigarette smoking are risk factors for the development of many non-communicable diseases (coronary heart disease, type 2 diabetes, osteoporosis, cancers) [2]. Dietary supplements represent an important source of essential nutrients, and millions of people worldwide have decided to use dietary supplements [3,4]. European Union (EU) Directive 2002/46/EC was the first dietary supplement regulatory act in the EU countries [5]. After joining the EU, Croatia developed regulations on dietary supplements in line with the EU Directive [6]. The German National Nutrition Survey II showed that 21% of young people used dietary supplements [7], including 66% of American students [8], 68% of Serbian students [9], and 43% of Malaysians students [10], while in Croatia, there are no existing data about dietary supplements use among young people. Young people are particularly vulnerable to the effects of poor nutrition, including nutrient deficiencies [11]. University students may differ from the general population in dietary supplement use [12] because of their specific university life, which includes studying, sports activities, smoking and alcohol consumption [13,14]. In addition, consumers often do not have enough knowledge about dietary supplements and their use and interaction with other food substances, and they often use them without consulting with healthcare professionals [15,16]. It is assumed that individuals who have a greater degree of education about health, such as medical students, have greater knowledge and are more selective regarding dietary supplement use than the general population. In addition, they can, in their future medical practice, have an influence on patient beliefs and uses of dietary supplements as well as an influence on the general population [17]. Many studies have explored differences in dietary supplements use, knowledge and attitudes between medical science and non-medical science students [9,10,18,19].

The aim of this study was to determine the prevalence of usage, knowledge and attitudes concerning dietary supplements among medical sciences and nonmedical sciences students from the University of Rijeka, Croatia. The second aim was to explore the association between dietary supplements intake and sex, knowledge, physical activity, body mass index (BMI) and university status.

## 2. Materials and Methods

### 2.1. Study Participants

This descriptive, cross-sectional study was conducted from January to May 2017 at the University of Rijeka, a medium-sized Croatian university. For the purposes of this study, we chose students from two departments with a similar number of students: medical sciences students (MSS) from the School of Medicine (SM) and nonmedical sciences students (NMSS) from the Faculty of Tourism and Hospitality Management (FTHM). A total of 2000 students from both departments and all study years were asked to participate in this study. They were asked to sign a consent form if they wished to participate in the study that was in accordance with the Declaration of Helsinki. The Ethics Committee of the Teaching Institute of Public Health of Primorsko-Goranska County approved the study protocol (7th Session of Teaching Institute of Public Health of Primorsko-goranska County held 2, December 2016). Out of 2000 students, 676 students did not wish to participate in the study, and 414 students returned incompletely filled out questionnaires. Therefore, a total of 910 students (45.5%) were incorporated in our study. According to the year of study at the time of data collection, participants were divided into freshmen (the first study year), juniors (second and third study year) and seniors (fourth and fifth study year).

### 2.2. Measurement

The questionnaire consisted of two sections. The first section contained sociodemographic and lifestyle characteristics of the study participants including sex, faculty, university status, BMI, smoking and physical activity habits. Physical activity habits were determined from questions about their physical activity level, and their responses were then categorized as 1 = no exercise, 2 = 1–2 times per week and 3 = 3 times per week or more. Body mass index (BMI) was calculated from body weight and body height and divided into four categories: underweight (<18.5 kg/m^2^), normal (18.5–24.99 kg/m^2^), overweight (25.00–29.99 kg/m^2^) and obese (≥30 g/m^2^). A second section of the questionnaire contained items asking about dietary supplement usage and about knowledge and attitudes regarding dietary supplements; for these items, we used questions from a validated questionnaire [19].

### 2.3. Statistical Analysis

For all statistical analyses, we used the data analysis software system Statistica, version 13 (TIBCO Software Inc., Palo Alto, CA, USA, 2017). The chi-square test for independence was used to detect relationships between group inclusion (MSS and NMSS) and their knowledge and attitude towards dietary supplements. The results for categorical variables are presented as *N* (%). To measure the association between variables we used Cramer’s V coefficient, as one variable was of nominal type. To investigate the nature of association we used the chi-square test. Differences were considered significant at *p* < 0.05 or less.

## 3. Results

Table 1 shows the sociodemographic and lifestyle characteristics of study participants. Overall, 73.8% of participants were women, and more than half of the study participants were nonmedical sciences students (60.2%). Junior students were in the majority (46.4%), followed by freshman (35.8%) and senior students (17.8%). Most of the participants had a normal BMI (78.9%) and were nonsmokers (60.7%), and more than half were not physically active (53.8%). The study showed that the prevalence of dietary supplement use among studied students was 30.5%.

**Figure 1:**
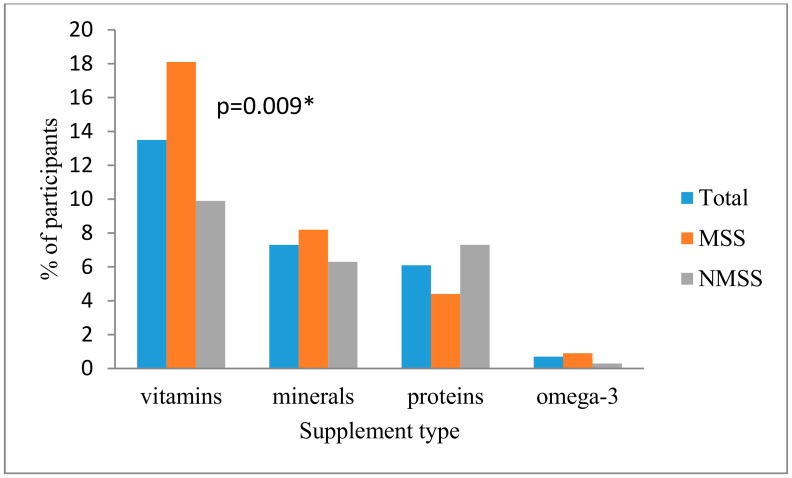
Types of dietary supplements consumed among the study participants (*N* = 910). Medical sciences students (MSS); Non- medical sciences students (NMSS).

The dietary supplements used most often were vitamins (18.0% in medical sciences students and 9.8% in nonmedical sciences students), followed by minerals and proteins in both groups (7.3% and 6.1%, respectively). The use of vitamins was significantly higher in medical sciences students than in nonmedical sciences students (*p* = 0.009) (Figure 1).

There was no significant difference in dietary supplement use between medical sciences students and nonmedical sciences students (*p* = 0.132) (Table 2). Our results showed that neither student group looked for professional medical help when taking dietary supplements (79.6%), and a small number of students participated in a health campaign on dietary supplements (11.2%). However, NMSS knew significantly less about what dietary supplements are (*p* < 0.001), and their level of belief that the use of dietary supplements is always safe was significantly higher (*p* = 0.009). In addition, both student groups thought that drugs, food or drink taken with dietary supplements did not interact (36.6%) (Table 2).

Regarding the information sources about dietary supplement intake, for all students, the internet (66.1%) was the most commonly used source of information, followed by healthcare professionals (33.2%), TV or journal advertisements (27.5%), friends and relatives (22.2%), professional literature (19.0%) and leaflets (18.2%) (Table 3). However, our results showed that medical sciences students significantly more often used product information leaflets (*p* < 0.001) and TV or newspapers (*p* < 0.001) as sources of information than did nonmedical sciences students. Nonmedical students significantly more often used relatives and friends as sources of information (*p* < 0.001). The most common reason for taking dietary supplements was to maintain good health (26.4%), followed by to ensure adequate nutrition (24.6%), to satisfy energy needs (23.7%), for weight loss (6.4%), to treat minor illnesses (5.1%) and to prevent diseases (4.8%). However, nonmedical sciences students had a significantly greater belief that supplements ensure adequate diet (*p* = 0.007), help weight loss (*p* = 0.010) and treat minor illnesses (*p* = 0.024). The results in our study showed that nonmedical sciences students agreed to a significantly greater degree that dietary supplements are necessary for all ages (*p* = 0.001), generally harmless (*p* = 0.026), prevent chronic diseases (*p* < 0.001), and, with regular use, prevent cancers (*p* < 0.001); these students also held the opinion that health personnel should promote supplement use (*p* = 0.002) (Table 3).

Table 4 shows association between supplement intake with sex, knowledge, physical activity, BMI and university status. Sex and knowledge are not associated, university status and physical activity were very weekly associated, while BMI is weakly associated with supplement intake, respectively.

On the basis of the values of the association coefficients, detailed analyses of the supplement intake association with variables (university status, physical activity and BMI) was performed. Statistically significant association was found only for BMI (*p* = 0.016) and physical activity (*p* = 0.050), while university status was not significantly associated with supplement intake (*p* = 0.090).

**Figure 2:**
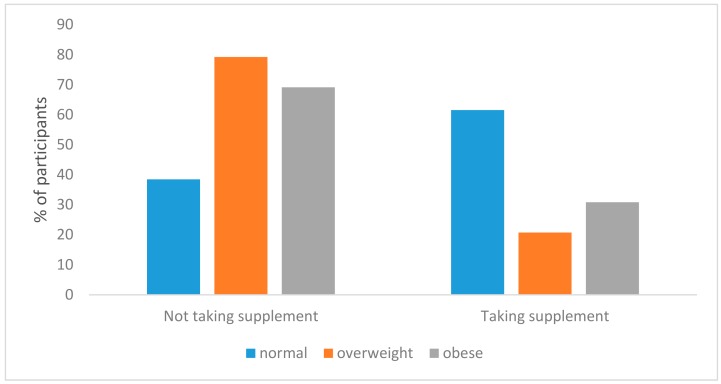
Supplement intake with respect to body mass index (BMI).

Results of the chi-square test showed that significantly greater proportion of the students who used supplements was in those students with normal BMI (61.5%) (*p* = 0.016). In a group of participants who took supplement, the percentage of the overweight students was 20.7%, while the percentage of the obese students was 30.9% (Figure 2).

**Figure 3:**
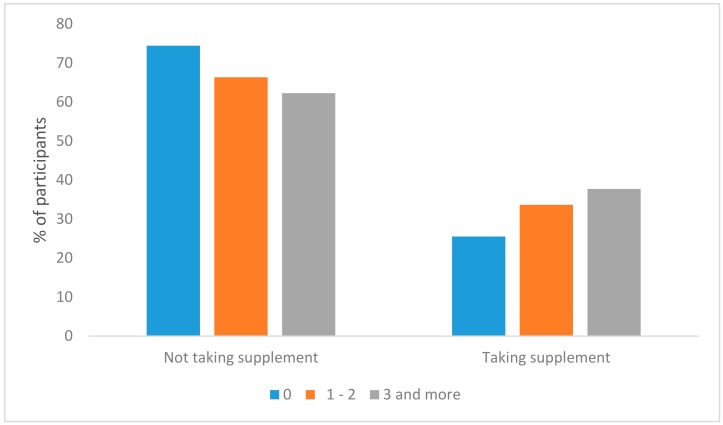
Supplement intake with respect to physical activity.

Results of the chi-square test showed that significant proportion of the students who used supplement was in the most physically active group (37.7%) (*p* = 0.050). The percentage of the least physically active students was 25.5% and the percentage of the moderate physically active students was 33.6% (Figure 3).

## 4. Discussion

To our knowledge, this study is the first in Croatia to document dietary supplement use in university students. The prevalence of dietary supplement use among studied university students was 30.5%, which is similar to results for students in Korea (31.3%) [18] and the Middle East (39%) [19] and to the results obtained by Sotoudeh et al. [18] for students in Tehran (33%) [20]. However, the usage prevalence among our students is lower than the prevalence among American (52%) [21], Australian (56%) [22], and Serbian students (68.1%) [23] and higher than in Jordanian (27.4%) [24] and Portuguese students (16%) [25]. Medical sciences students (33.1%) were slightly higher users of dietary supplements than were nonmedical sciences students (28.8%), which is in accordance to similar studies [19,26]. In line with other studies of dietary supplements, for our students, the most popular dietary supplements were vitamins (13.8%) [9,10,18]. In addition, many similar studies reported multivitamins and multivitamin-mineral combinations as the most frequently used dietary supplements in the student’ population [24,27,28].

Our research showed that medical science students knew what dietary supplements are, and that their use is not always safe, which was significantly higher, compared to NMSS, (*p* < 0.001) and (*p* = 0.009), respectively. This is expected to be due to the pharmacology course and other medical courses undertaken by these students, which give better knowledge about the risks of dietary supplement use. Similar findings have been reported in other studies [10,19,29]. In our study, the internet has the strongest influence (66.1%) for all participants regarding decisions to take supplements, while healthcare professionals were second (33.2%). Studies dealing with dietary supplement usage among students [10,30] have reported that family, friends and doctors were the most important sources of dietary supplement information, while among our studied students, the internet was the most commonly used source of information, followed by healthcare professionals. The internet provides a great deal of information about dietary supplements, but it can also provide unreliable and unverified data that can lead to wrong decisions about dietary supplements use. A study like this provides useful information concerning the usage and prevalence of dietary supplements and about the attitudes and reasons for their use among young people, which is valuable support for public health actions. The prevalent use of the internet as a source for dietary supplements usage rather than health professionals as a trusted source is very useful information, especially considering the data showing that a large proportion of students (79.6%) ask for no medical professional help when they take a dietary supplement. The decision about trusted sources should be revised in the young population to support better health choices. Medical students have medical courses, which give them basic knowledge about nutrition and dietary supplements. This is why we expected them to have better knowledge regarding dietary supplements, especially when answering questions like “do dietary supplement interact with food and drink” or “weather medical help is needed before consuming dietary supplement”. The main reasons stated for students to use dietary supplements was to maintain good health (26.4%), to ensure adequate nutrition (24.6%) and to satisfy their energy needs (23.7%). Those results were similar to those of other studies that reported similar reasons for using dietary supplements [10,21,19]. Although there was no significant difference between dietary supplement use between medical and nonmedical science students, medical science students had significantly less positive attitudes about health benefits of supplements than nonmedical science students did, which is in line with other studies [10,29].

A significantly higher proportion of students with normal BMI and those who are the most physically active use dietary supplement. Since the majority of the students use dietary supplements to maintain good health, it can be suggested that healthy lifestyle is related to physical activity and healthy diet.

The study has some strengths in addition to being the first of its kind in Croatia; it provides information useful for public health messaging, and we used a validated questionnaire and a reliable sample of the student population. On the other hand, the study lacks more accurate information about dietary supplement usage besides those covered in the study, about dietary supplements producers and products, and about other sociodemographic information; this represents a study limitation. Further study will be needed to widely investigate those connections that are useful for public health programs.

## 5. Conclusions

In our study, dietary supplement use among university students was relatively low. Use of the internet rather than of health professionals as a trusted information source should be revised among this young population. It is very important for medical students to understand the role of dietary supplements and their potential harmful interaction with food and drinks in order to become trusted sources of information. Although the evidence-based data on the use of dietary supplements is still weak, these study results could serve as support for medicine faculties to improve their curriculum to encourage better and safe use of dietary supplements and to support the development of public health messages aimed at the wise and safe use of dietary supplements.

## Figures and Tables

**Table 1:** Sociodemographic and lifestyle characteristics of study participants (*N* = 910).

Variables		*N*	%
Sex	Men	238	26.2
	Women	672	73.8
Faculty	Medical sciences students (MSS)	362	39.8
	Nonmedical sciences students (NMSS)	548	60.2
University status	Freshmen	326	35.8
	Juniors	422	46.4
	Seniors	162	17.8
BMI (kg/m^2^)	Underweight	60	6.6
	Normal	718	78.9
	Overweight	106	11.6
	Obese	26	2.9
Smoking	Yes	358	39.3
	No	552	60.7
Physical activity (times/week)	0	490	53.8
	1–2	198	21.8
	3 and more	222	24.4
Take dietary supplements	Yes	278	30.5
	No	632	69.5

**Table 2:** Questionnaire used to determine knowledge, attitudes and practices regarding dietary supplements in study participants from medical and nonmedical sciences faculties (*N* = 910) (*N* (%)).

Questions	Total (*N* = 910)	MSS (*N* = 362)	NMSS (*N* = 548)	*p*-Value
Do you know what dietary supplements are?				
Yes	834 (91.6)	338 (93.4)	325 (59.3)	<0.001 **
No	46 (5.1)	20 (5.5)	197 (36.0)	<0.001 **
Do not know	30 (3.3)	4 (1.1)	26 (4.7)	<0.001 **
Have you attended any health campaigns/workshops on dietary supplements?				
Yes	102 (11.2)	30 (8.3)	72 (13.1)	0.108
No	790 (86.8)	322 (88.9)	468 (85.4)	0.274
Do not know	18 (2.0)	10 (2.8)	8 (1.5)	0.329
Do you use any dietary supplements?				
Yes	278 (30.5)	120 (33.1)	158 (28.8)	0.132
No	632 (69.5)	242 (66.9)	390 (71.2)	0.328
I always look for professional medical help when taking a dietary supplement.				
Yes	134 (14.7)	54 (14.9)	80 (14.6)	0.924
No	724 (79.6)	290 (80.1)	434 (79.2)	0.813
Do not know	52 (5.7)	18 (5.0)	34 (6.2)	0.580
Do you think the use of dietary supplements is always safe?				
Yes	68 (7.5)	32 (8.8)	85 (15.5)	0.009 *
No	548 (60.2)	178 (49.2)	321 (58.6)	<0.001 **
Do not know	294 (32.3)	152 (42.0)	142 (25.9)	<0.001 **
Do you think that drug, food or drinks taken with dietary supplements might interact?				
Yes	219 (24.1)	76 (21.0)	143 (26.1)	0.166
No	333 (36.6)	146 (40.3.)	187 (34.1)	0.197
Do not know	358 (39.3)	140 (38.7)	218 (39.8)	0.825

* *p* < 0.05, ** *p* < 0.001. Medical sciences students (MSS); Non-medical sciences students (NMSS).

**Table 3:** Source of dietary supplements information and reasons for taking dietary supplements among the study participants from medical and nonmedical sciences faculties (*N* = 910) (*N* (%)).

Source	Total (*N* = 910)	MSS (*N* = 362)	NMSS (*N* = 548)	*p*-Value
Healthcare professionals	302 (33.2)	122 (33.7)	180 (32.8)	0.849
Internet	602 (66.1)	224 (61.9)	378 (69.0)	0.017
Product information leaflets	166 (18.2)	108 (29.8)	58 (10.6)	<0.001 **
Professional literature	172 (19.0)	64 (17.7)	108 (19.7)	0.588
Friends and relatives	202 (22.2)	48 (13.3)	154 (28.1)	<0.001 **
TV or journal advertisements	250 (27.5)	132 (36.5)	118 (21.5)	<0.001 **
Reasons				
Maintain good health	240 (26.4)	84 (23.2)	156 (28.5)	0.212
Ensure adequate nutrition	224 (24.6)	64 (17.7)	160 (29.2)	0.007 *
Treat minor illnesses	46 (5.1)	8 (2.2)	38 (6.9)	0.024 *
Satisfy energy needs	216 (23.7)	76 (21.0)	140 (25.5)	0.263
Prevent diseases	44 (4.8)	10 (2.8)	34 (6.2)	0.090
Weight loss	58 (6.4)	10 (2.8)	48 (8.6)	0.010 *
No specific reason	12 (1.3)	4 (1.1)	8 (1.5)	0.745
Attitudes that study participants agreed with				
Dietary supplements are necessary for all ages	118 (13.0)	38 (32.2)	80 (67.8)	0.001 *
Dietary supplements are generally harmless	194 (21.3)	78 (40.2)	116 (59.8)	0.026 *
Regular use of supplements prevents chronic diseases	110 (12.1)	32 (29.1)	78 (70.9)	<0.001 **
Dietary supplements can prevent cancers	74 (8.1)	16 (21.6)	58 (78.4)	<0.001 **
Health personnel should promote supplement use	146 (16.0)	50 (34.2)	96 (65.8)	0.002 *

* *p* < 0.05, ** *p* < 0.001. Medical sciences students (MSS); Non-medical sciences students (NMSS).

**Table 4:** Association between Supplement intakes with: sex, knowledge, physical activity, BMI and university status.

Variables	Cramér’s V
Sex	0.0473
Knowledge	−0.0067
Physical activity	0.1147
BMI	0.1350
University status	0.1028
